# Metastatic colo-rectal cancer: real life experience from an Indian tertiary care center

**DOI:** 10.1186/s12885-021-08398-z

**Published:** 2021-05-28

**Authors:** Vinod Sharma, Atul Sharma, Vinod Raina, Deepak Dabkara, Bidhu Kalyan Mohanti, N. K. Shukla, Sushmita Pathy, Sanjay Thulkar, S. V. S. Deo, Sunil Kumar, Ranjit Kumar Sahoo

**Affiliations:** 1 Department of Medical Oncology, Dr BRA IRCH, All India Institute of Medical Sciences (AIIMS), New Delhi, India; 2 Department of Medical Oncology, Tata Memorial Center, Kolkata, India; 3 Department of Radiotherapy, Dr BRA IRCH, All India Institute of Medical Sciences (AIIMS), New Delhi, India; 4 Department of Surgical Oncology, Dr BRA IRCH, All India Institute of Medical Sciences (AIIMS), New Delhi, India; 5 Department of Radiodiagnosis, Dr BRA IRCH, All India Institute of Medical Sciences (AIIMS), New Delhi, India

**Keywords:** Metastatic, Colorectal cancer, Real life experience, India

## Abstract

**Background:**

No data exist for the long-term outcome of metastatic colorectal cancer (mCRC) from the Southern part of Asia. The primary objective of the study is to evaluate the survival outcome of mCRC from an Indian tertiary care center. The study also aims to highlight the treatment pattern practiced and the unique clinico-pathologic characteristics.

**Methods:**

This is a single-center retrospective observational study done at a large referral tertiary care center in North India. All patients with synchronous or metachronous mCRC who received at least one dose of chemotherapy for metastatic disease, registered between 2003 to 2017 were included. Primary outcome measures were overall survival and progression-free survival and prognostic factors of overall survival. Descriptive analysis was done for the clinicopathological characteristics and treatment patterns. Kaplan Meier method for overall survival and progression-free survival. Cox regression analysis was performed for the determination of the prognostic factors for overall survival.

**Result:**

Out of 377 eligible patients, 256 patients (68%) had de novo metastatic disease and the remaining 121 (32%) progressed to metastatic disease after initial treatment. The cohort was young (median age, 46 years) with the most common primary site being the rectum. A higher proportion of signet (9%) and mucinous histology (24%). The three common sites of metastasis were the liver, peritoneum, and lung. In the first line, most patients received oxaliplatin-based chemotherapy (70%). Only 12.5% of patients received biologicals in the first-line setting. The median follow-up and median overall survival of study cohort were 17 months and 18.5 months. The factors associated with poor outcome for overall survival on multivariate analysis were ECOG performance status of > 1, high CEA, low albumin, and the number of lines of chemotherapy received (< 2).

**Conclusion:**

The outcome of mCRC is inferior to the published literature. We found a relatively higher proportion of patients with the following characteristics; younger, rectum as primary tumor location, the signet, and mucinous histology, higher incidence of peritoneum involvement. The routine use of targeted therapies is limited. Government schemes (inclusion of targeted therapies in the Ayushman scheme), NGO assistance, and availability of generic low-cost targeted drugs may increase the availability.

## Introduction

Colorectal cancer (CRC) is important cause of cancer related morbidity and mortality. Worldwide, it stands second for cancer related mortality [1]. In South Central Asia (including India), the incidence and mortality of CRC is comparatively lower than the western world. However, half of the world cancer occur in Asia. Current literature suggest rise in the incidence of CRC in Asian countries (China, Singapore, Japan, Sri Lanka) [2, 3]. Fluoropyrimidine based combination therapy including either oxaliplatin (FOLFOX with various modifications, XELOX) or Irinotecan (FOLFIRI, XELIRI) plus targeted therapy is the current standard of care for metastatic colorectal cancer worldwide as incorporated by various guidelines [4]. The median overall survival has improved successively from 8 to 10 months with single agent drug to 18–24 months with chemotherapy doublets, with reaching up to 34 months with addition of biologics [5].

However, these advancements have not reached the routine day to day clinical management of metastatic CRC in many parts of the world. As per Globocan 2018, in India, CRC accounts for the sixth and seventh cause of cancer related disease burden and cancer related death respectively [1]. Despite a low prevalence, according to the National cancer registry programme, the expected number of CRC in India by 2020 will reach close to 100,000 [6]. This number is huge and it poses a great challenge to the treating physicians. In India, most patients are not insured and have to purchase costly drugs out of pocket. No established screening colorectal cancer programme exist. All CRC diagnosis are symptomatic at presentation.

The long term outcome and treatment pattern from Indian sub continents has not been published. Most of the studies has been focused on the clinico pathological characteristics including all stages and are limited by having small number of patients [7–9].

The primary goal of our study is to study the patient characteristics, prognostic factors, pattern of care using first and second line chemotherapy and associated prognostic outcome among patients with metastatic CRC treated at our institutions over the last 15 years. This is the first study from Indian sub-continent highlighting the treatment outcome of metastatic colorectal cancer from a tertiary care cancer centre.

## Materials and methods

We conducted a retrospective analysis of patients with metastatic colorectal cancer, registered between January 2003 to December 2017. The study protocol was approved by the institutional ethical committee (AIIMS, All India Institute of Medical Sciences, New Delhi). Standard guidelines and regulations were followed for the study. The institute ethical committee (AIIMS, All India Institute of Medical Sciences, New Delhi) also exempted the study from the informed consent. All files numbers were extracted from the patients record database - computer based system and in hospital day care record system to ensure screening for all patients with diagnosis of colorectal cancer. All files were screened and subsequently patients who received treatment for metastatic disease were retrieved. Only patients who received at least 1 cycle of chemotherapy with histopathological diagnosis of colorectal cancer were enrolled into the study. Patients treated with best supportive care and palliative RT only were not included.

Patient and treatment characteristics were filled in pre specified performa. The main objective of the study was to determine the overall survival and the prognostic factors affecting the outcome measures. Overall survival was calculated from the date of institution of first line chemotherapy for metastatic disease to the date of last follow up in surviving patients or date of death from any cause. The secondary objective was to determine the PFS and the prognostic factors determining it. Progression free survival (PFS) was calculated from the date of institution of first line chemotherapy for metastatic disease to the date of progressive disease or death from any cause whichever is first. Patients who were lost to follow up, were contacted telephonically for survival status. Data for surviving patients was censored on 31st December 2018. Response assessment was done first after at least 3–4 cycles (roughly 2 months) unless clinically indicated and then attempted to be every 4–6 months. Response assessment was done either using CECT (contrast enhanced computed tomography) or PET CT (positron emission tomography computed tomography) depending upon the availability of modality. Overall response rate (ORR) included partial response and complete response. Clinical benefit rate (CBR) included the sum of complete response, stable disease and partial response. All statistical computations were done using STATA software version 13. Descriptive analysis were done for baseline characteristics. Time to event analysis was done using Kaplan-Meier survival curve estimates. Cox regression analysis was performed for prognostic factor.

A number of chemotherapy regimen in standard dosing form were used over the last two decade. Standard doses of chemotherapy doublet were administered including FOLFIRI, CapeOx / XELOX (Capecitabine doses - 1 g/m2/BD for 2 weeks every 3 weekly), IFL etc. For initial few years, an institutional modified FOLFOX protocol constituting oxaliplatin 85 mg/m2 on day 1 and leucovorin (LV) 200 mg/m2, 5-FU (5- Fluorouracil) 400 mg/m2 push, 5-FU 600 mg/m2 over 8 h, each on day 1 and 2 was instituted. Later the modified FOLFOX-6 regimen was used. Left side tumor included rectum, sigmoid colon, descending colon, splenic flexure of colon. Right sided tumor included hepatic flexure of colon, caecum and full length ascending colon.

## Result

### Patient characteristics

**Fig. 1:**
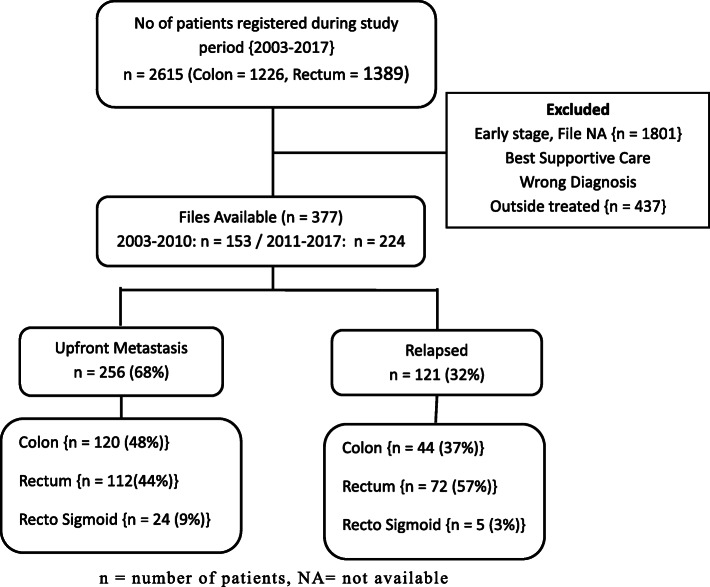
Patient disposition Diagram. n = number of patients, NA = not available

Between 2003 and 2017, a total of 2615 patients with the diagnosis of colorectal cancer were registered. Out of these, 377 were found eligible for the study enrollment. A large number of patients met the exclusion criteria (n 2238). The various reasons for exclusion were early-stage disease (most common), wrong diagnosis, best supportive care, received outside treatment, as enlisted in the patient disposition flow diagram (Fig. 1). Two hundred and fifty six patients (68%) presented with upfront metastatic disease and remaining 121 (32%) progressed to metastatic disease after initial treatment.

The cohort was relatively young with median age of 46 years (range, 11 to 82 years). Six patients had age of less than 18 years. The ratio of male to female was 1.4:1 (Table 1). Young adults (≤ 40 years) constituted 38% of study population. The median duration of symptoms in patients with up front metastatic disease was 6 months (range, 3–10 months). In relapsed cases, the median time to metastasis (time from treatment initiation for localized disease to relapse) was 15.5 months (range, 8–27 months). Most patients received systemic therapy with palliative intent and only few underwent curative resection. Three fourth of patient had ECOG PS of 0–1. A positive family history of cancer was present in 12% of cases, with 6% being gastrointestinal and 2% were CRC.

**Table 1:** Baseline characteristics, Pathology

Variable	Number (%) of patients
Median Age (range) years	46 (11–82)
Gender (Male / Female)	219 (58%) / 158 (42%)
Age ≤ 40 yrs. and ≥ 65 yrs.	144 (38%) / 33 (8.8%)
Median duration of symptoms, months	6 (3–10)
ECOG performance status (*n* = 320)	
0–1	223 (72%)
2–3	89 (28%)
F/h of cancer / Gastrointestinal cancer (n = 320)	38 (12%) / 17 (6%)
Upfront Metastasis / Relapsed post adjuvant therapy	256 (68%) / 121 (32%)
Presenting symptoms (Up front metastasis)	
Abdominal pain or pain during defecation	168 (65%)
Bleeding Per rectum	148 (57%)
Altered bowel habits	136 (52%)
Weight loss	70 (27%)
Obstruction	34 (13%)
Laboratory parameters (median)	
Hemoglobin (*n* = 321)	10.7 (9.3–12.3)
Platelets (*n* = 320)	272,000 (18600–336,000)
Total leucocyte count (*n* = 320)	7850 (6400–9500)
Albumin (*n* = 317)	3.9 (3.6–4.4)
CEA (ng/ml) (median, *n* = 293)	22 (5–117)
High CEA level (> 5 ng/ml)	228 (78%)
High Serum alkaline phosphatase (*n* = 307)	11 (3.6%)
Grade (*n* = 342)	
Well / Moderately	323 (94%)
Poorly differentiated	19 (6%)
Morphology (*n* = 325)	
Adenocarcinoma NOS	216 (66%)
Signet ring	30 (9.2%)
Mucinous	78 (24%)
Adenosquamous	1 (0.3%)

*CEA* carcinoembryonic antigen, *NOS* not otherwise specified

### Tumor Characteristics (Symptom, Metastasis, Side, Pathology, and Mutation status)

Most common symptom was abdominal pain or pain during defecation (65%) followed by bleeding per rectum (57%), altered bowel habits (52%), weight loss (27%) and obstruction (13%). Most of the tumors were well to moderately differentiated (94%) with one case of adenosquamous. A high proportion of patient had mucinous (24%) morphology followed by signet (9.2%). Proportion of left side and right side tumor was 75 and 20% (Table 2). The most common primary site was rectum (47%) followed by sigmoid colon (17%), caecum (8%) and ascending colon (8.7%). The mean number of the site of organ metastasis were 1.1 (SD ± 0.42). The common sites of metastasis were liver (43%), peritoneum (31%), lung (18%) followed by ovary (13%) and bone (5%). Nearly two third of the patients with liver metastasis had more than 3 lesions. Mean number of organs involved at diagnosis were 1.1 (SD ± 0.04). The proportion of patients with one, two and three organ involvement was 76, 21 and 2.4% respectively. Mutational analysis was done in limited number of patients, the mutation rate were {KRAS (22/70; 33%), NRAS (1/33;3%), BRAF (2/30; 7%), MSI (8/32; 25%).

**Table 2:** Sidedness, Metastasis pattern

Variable	Number (%) of patients
Right Side	75 (20%)
Caecum	30 (8%)
Ascending colon	33 (8.7%)
Hepatic flexure	12 (3.1%)
Left side	285 (76%)
Splenic flexure	3 (0.8%)
Descending colon	13 (3.5%)
Sigmoid colon	64 (17%)
Recto sigmoid	29 (7.7%)
Rectum	176 (47%)
Transverse Colon	15 (4%)
Metastasis	
Liver	161 (42.7%)
Peritoneum	117 (31%)
Lung	68 (18%)
Non regional lymph nodes	57 (15%)
Ovary	49 (13%)
Bone	19 (5%)
Skin	3 (0.8%)
Adrenal	6 (1.7%)
Number of metastatic site (Mean ± SD)	1.1 ± 0.04
Number of organ involved	
1	287 (76%)
2	80 (21%)
3	10 (2.6%)
No of liver lesion > 3	101 (63%)

For 2 cases the primary site was not documented

### Treatment outcome

All these patients (377 patients) received at-least one dose of chemotherapy for metastatic disease. Second line chemotherapy was administered in 33% (*n* = 124) of study population. Oxaliplatin and irinotecan based chemotherapy was the main regimen used in 1st and 2nd line chemotherapy (71% vs 59%) respectively. The various chemotherapy regimen used in 1st line were CapeOx followed by FOLFOX, FOLFIRI, CAPIRI etc (Table 3). Similarly in second line, the most common regimen was FOLFIRI (37%) followed by CAPIRI (22%), FOLFOX (15%) and others. Median number of cycles received were 6 in both 1st and 2nd line therapy. Most patients received doublet regimen except few during early part of 2003–2005 when single agent chemotherapy was given. The type of first line chemotherapy doublet remained unchanged over the study duration. About 12.5% of patients received biologicals in first line. The proportion of patient who received biological agents in first, second and third were 12.5, 31.5 and 48% respectively (Table 4). The median number of chemotherapy lines given was 1 (range, 1 to 5). A documented response was available in 76 and 67% of patients respectively in first and second line. In first line setting, the progressive disease, overall response rate and stable disease were seen in 40, 34 and 26% cases respectively. Similarly for second line, the overall response rate, stable disease, progressive disease were 22, 32, and 37% respectively (Table 5).

**Table 3:** Chemotherapy regimen given in first and second line setting

Chemotherapy Line	Regimen	N (%)
First line	CapeOx	166 (44%)
	FOLFOX	75 (20%)
	FOLFIRI	49 (13%)
	CAPIRI	34 (9%)
	Capecitabine	11 (3%)
	FOLFOXIRI	5 (1.3%)
	Others (FUFA, FLOX, IFL)	37 (10%)
Second line *N* = 124	FOLFIRI	47 (37%)
	CAPIRI	27 (22%)
	FOLFOX	19 (15%)
	CapeOx	14 (11.3%)
	Others (IFL, Capecitabine, UFT, FOLFOXIRI, Irinotecan)	17 (14%)

CapeOx, Capecitabine, oxaliplatin; FOLFOX, 5-FU, LV, oxaliplatin; FOLFIRI, 5-FU, LV, Irinotecan; CAPIRI, Capecitabine, Irinotecan; FOLFOXIRI, 5-FU, LV, oxaliplatin, Irinotecan; FUFA, 5-FU, LV; FLOX, 5-FU, oxaliplatin; IFL, 5-FU, Irinotecan, UFT -Uracil

**Table 4:** Chemotherapy lines and drug used

Chemotherapy line	Oxaliplatin	Irinotecan	VEGF MoAB	EGFR MoAB	CT alone	CT + MoAB
1st (100%) *n* = 377	71%	26%	4.5%	7.9%	87.5%	12.5%
2nd (33%) *n* = 124	26.4%	59%	19%	12.6%	68.5%	31.5%
3rd (8.4%) *n* = 32	48%	29%	30%	17%	52%	48%

*VEGF* Vascular endothelial growth factor, *MoAB* monoclonal antibodies, *CT* chemotherapy, *EGFR* Epidermal growth factor receptor

**Table 5:** Response rate to first and second line

Response category	First line (%, n 310)	Second line (%, n 83)
ORR^a^	34%	22%
Stable Disease	26%	32%
CBR^b^	60%	54%
Progressive Disease	40%	37%

^a^Overall response rate

^b^Clinical benefit rate

The toxicity recording was not up to the mark. The major grade 3–4 toxicity seen with first line regimens was anemia (13.4%), leucopenia (5%), diarrhea (5.6%), thrombocytopenia (5.6%). The all grade peripheral neuropathy was 10%, with grade 3–4 being only seen in 1.6% of cases (data not shown).

**Fig. 2:**
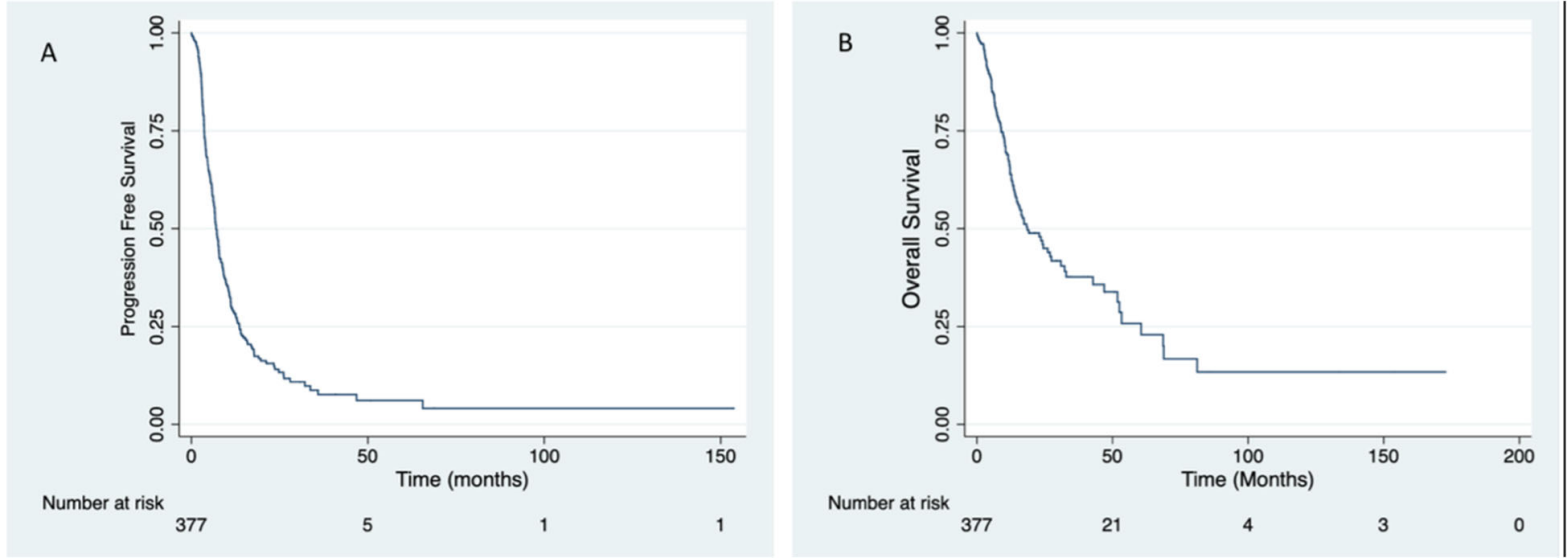
Kaplan-Meier survival estimates: **A** Progression Free Survival (7.13 months); and **B** Overall Survival (18.5 months)

With the median follow up was 17 months, the median progression free survival (PFS) and overall survival (OS) for the study cohort were 7.13 months and 18.5 months respectively (Fig. 2). The overall survival rate at 2 years, 3 years and 5 years of the entire cohort was 45, 37 and 23% respectively. The median overall survival for patients who received more than 1 line of chemotherapy was 23.4 months (95% CI; 14–47 months). For progression free survival, on univariate analysis significant factors were ECOG 0–1, Hemoglobin, CEA, hypoalbuminemia. On multivariate analysis only 2 factors came out to be significantly associated with poor PFS; high CEA and ECOG > 1 (Table 6). On univariate analysis for overall survival four factors including ECOG Performance status (PS) 0–1, the number of lines of chemotherapy, high CEA and hypoalbuminemia came out to be significant. On multivariate analysis using Cox regression, all these factors remained significant (*p* < 0.05) (Table 7). Use of biological therapies as covariate for overall survival and progression free survival was not significant.

**Table 6:** Univariate and multivariate analysis of different prognostic factors for progression free survival

Variable	n	Univariate Analysis			Multivariate analysis		
		Hazard Ratio (HR)	p	95% CI	HR	p	95% CI
Sex							
Female	158	1.03	0.787	0.78–1.36			
Male	219						
Elderly							
> 65 yrs	33	0.75	0.243	0.46–1.2			
≤ 65 yrs	344						
Young							
≤ 40 yrs	144	1.3	0.051	0.99–1.73			
> 40 yrs	233						
**ECOG**							
**≥ 2**	**89**	**1.58**	**0.005**	**1.15–2.17**	**1.5**	**0.032**	**1.03–1.6**
**0–1**	223						
Upfront							
Metastasis	256	1.22	0.182	0.91–1.63			
Relapsed	121						
Hb (g/dl)							
< 10.8	165	1.48	**0.006**	1.12–1.96	1.14	0.44	0.8–1.6
≥ 10.8	156						
**CEA**							
**High**	**228**	**1.66**	**0.008**	**1.14–2.42**	**1.80**	**0.005**	**1.2–2.7**
**Normal**	65						
Albumin(g/dl)							
< 3.5	54	1.49	**0.043**	1.01–2.20	1.26	0.309	0.8–1.96
≥ 3.5	263						
Sidedness							
Left	285	1.06	0.71	0.76–1.48			
Right	75						
Grade							
Poorly	19	1.60	0.12	0.87–2.9			
Moderately / Well	304						
Morphology							
Signet / Mucinous	108	1.16	0.33	0.86–1.56			
Adenoca NOS	216						
SAP							
High	11	1.67	0.216	0.73–3.82			
Normal	296						
No of organs involved							
≥ 2	90	1.3	0.09	0.95–1.80			
1	287						
Liver metastasis							
Yes	161	1.08	0.573	0.82–1.42			
No	216						
Peritoneum metastasis							
Yes	117	1.13	0.40	0.84–1.52			
No	300						
No of liver Metastasis							
> 3	89	1.17	0.475	0.75–1.85			
≤ 3	51						
CT							
Irinotecan	90	1.25	0.162	0.91–1.72			
Oxaliplatin	247						

*CT* Chemotherapy, *Hb* hemoglobin, *ECOG* Eastern Cooperative Oncology group, *CEA* carcinoembryonic antigen, High CEA > 5 ng/ml, *SAP* Serum Alkaline Phosphate, *CI* Confidence interval, *HR* Hazard Ratio

**Table 7:** Univariate and multivariate analysis of different prognostic factors for overall survival

Variable	n	Univariate Analysis			Multivariate analysis		
		HR	p	95% CI	HR	p	95% CI
Sex							
Female	158	1.06	0.716	0.74–1.52			
Male	219						
Elderly							
> 65 yrs	33	0.7	0.298	0.38–1.33			
≤ 65 yrs	344						
Young							
≤ 40 yrs	144	1.31	0.91	0.83–1.89			
> 40 yrs	233						
ECOG							
≥ 2	**89**	1.79	**0.005**	1.18–2.70	2.0	**0.003**	1.3–3.3
0–1	223						
Upfront							
Metastasis	259	1.23	0.297	0.83–1.81			
Relapsed	118						
Hb(g/dl)							
< 10.8	165	1.45	0.076	0.96–2.04			
≥ 10.8	156						
**CEA**							
**High**	**228**	**1.94**	**0.019**	**1.16–3.38**	**2.47**	**0.004**	**1.33–4.6**
**Normal**	65						
**Albumin**(g/dl)							
**< 3.5**	54	**2.48**	**0.001**	**1.56–3.95**	**1.71**	**0.045**	**1.0–2.9**
**≥ 3.5**	263						
Sidedness							
Left	285	0.72	0.124	0.48–1.09			
Right	75						
Grade							
Poorly	19	2.11	0.077	0.92–4.85			
Moderately / Well	304						
Morphology							
Signet / Mucinous	108	1.43	0.072	0.96–2.11			
Adenoca NOS	216						
SAP							
High	11	1.57	0.442	0.49–4.98			
Normal	296						
No of organs involved							
≥ 2	89	1.43	0.085	0.95–2.15			
1	287						
Liver metastasis							
Yes	161	0.98	0.90	0.68–1.39			
No	216						
Peritoneum metastasis							
Yes	118	1.3	0.155	0.90–1.92			
No	259						
Number of liver metastasis							
> 3	89	1.15	0.712	0.62–1.99			
≤ 3	51						
1st Line							
Irinotecan	90	1.1	0.54	0.76–1.6			
Oxaliplatin	247						
Number of lines of CT							
> 1	124	0.48	**0.001**	0.32–0.7	0.47	**0.001**	**0.3–0.74**
1	253						

*CT* Chemotherapy, *Hb* hemoglobin, *ECOG* Eastern Cooperative Oncology group, *CEA* carcinoembryonic antigen, High CEA > 5 ng/ml, *SAP* Serum Alkaline Phosphate, *CI* Confidence interval, *HR* Hazard Ratio

## Discussion

The study finds the presence of relatively younger cohort, high rate of rectal cancer, peritoneal metastasis and signet and mucinous histology. The poor performance status, low albumin, high CEA were predictors of poor survival outcome.

The median age at presentation of 46 years is in wide variance from the developed world where the median age is in 6th decade [10]. A number of studies done over the last 2 decades from India also suggest a similar median age [7–9]. A simple reason for the variance could be due to age pyramid of our country where most (80%) of the population is under 50 years of age and low mean life years (67–69 years) [11]. The median duration of symptoms in our patients was 6 months (range, 3–10 months) similar to previous studies [9, 12]. More than half of the patients presented with pain, bleeding per rectum, altered bowel habits. A significant proportion of patients carried family history of cancer (12%) with 6% being gastrointestinal (2% colon) [10].

Rectum was the most common site followed by the sigmoid colon, caecum, ascending colon and others. This is in contrast to developed countries, where rectum represents only 25–30% of all cases [12, 13]. Similar experience was observed in other studies across the country [9, 14]. Adenocarcinoma-not otherwise specified is the most common histology (90%) followed by mucinous and signet [15]. We found a significantly high percentage of patients with signet (9.2%) and mucinous histology (24%). A number of small studies done over the last 2 decade has repeatedly shown the similar observations [prevalence of signet ring morphology (13–19%) and mucinous tumors (16–31%)] [9, 16]. We found higher rate of peritoneal metastasis (31%) compared to 16–28% reported by Hugens et al. [15]. The high frequency of signet morphology in our cohort is probably the reason for the high frequency of peritoneal involvement. The frequency of KRAS (30%) mutations were less than the internationally reported figures [17, 18]. A relatively lower rate of KRAS mutation positivity reported across India (23–42%) [19, 20]. These studies were heterogenous and large database will be needed to conclude definitively about the incidence of mutations in Indian population. KRAS mutation variation is also seen across various European countries (33.7–54.1%) [21].

The objective response rate and progression free survival for first line chemotherapy regimen was 34% and 7 months similar to previous reported studies [22–24]. Overall survival and progression free survival in our study was 18.5 months and 7.13 months with a median follow up 17 months (Fig. 2). Tounigard et al. and Colucci et al., also reported a somewhat similar median overall survival of 21.5 months and 15 months with chemotherapy alone respectively. Only one third patients (33%) received second line chemotherapy regimen. The exact cause for the limited use of second-line chemotherapy in the study cohort is not clear. However, the day-to-day practice suggests that it could be related to multiple factors. Most of the patients were not insured; out-of-pocket purchase is routine for chemotherapy administration. Cancer services are limited to a small number of government-aided hospitals providing affordable chemotherapy services. People often have to travel long distances to get cancer care, which affects compliance and unplanned interruption. The limited use of biologicals also marks similar findings. The other reason could be aggressive biology due to the high proportion of EOCRC (early onset colo-rectal cancer) patients. However, the overall response rate and progression-free survival were comparable to published literature with chemotherapy doublets.

We studied 18 variables as potential predictive factor for overall survival, on multivariate analysis poor ECOG at baseline (≥2), high CEA, only one line of chemotherapy and hypoalbuminemia were associated with poor overall outcome. Poor ECOG at baseline has been proved to be a predictor of poor outcome in a number of studies [25, 26]. ECOG is one of the few important factors of Kohne prognostic and GERCOR score [26, 27]. High CEA has been variably reported as a poor prognostic factor in previous studies [28, 29]. Stelzner et al. in a retrospective study of 186 patients with synchronous metastasis found poor PS and high CEA associated with poor outcome [25]. High baseline CEA has been found to be associated with poor outcome in a prospective randomized trial [22]. However in prognostic score model where a large number of poor outcome factors were studied, CEA lost its relative significance. The median overall survival of metastatic CRC has improved over the last 2 decade with the use of doublet chemotherapy, biologicals and increased number of drugs for later lines. In today’s era, the treatment of CRC is considered as continuum of care [30]. The progression free survival for 1st and 2nd line therapy however remained more or less unchanged over the last one decade; but the sequential use of all effective drugs has improved the outcome reaching the median overall survival in current trials to 30–32 months. The best outcome can be achieved by providing the benefit of all active drugs in patient care. This was recently emphasized by Grothey et al. In a study of eleven phase III trials in advanced colorectal cancer, multivariate analysis showed association of overall survival with more number of drugs exposed [31]. In our study those who received more than one line of chemotherapy had better outcome with median overall survival of 23.4 months (95% CI; 14–47 months).

Sidedness has been recently identified to be significant prognostic factor for survival outcome. Right and left defined variably has been shown to be associated with survival difference. In CALGB 80405 study, the median OS was significantly better in left side cancers. The overall survival of the left and right side tumor with bevacizumab (32.6 months and 29.2 months) and cetuximab (39.3 months and 13.6 months) was remarkably different respectively [32]. The magnitude of difference appeared to be more pronounced with the use of anti-EGFR inhibitors. In FIRE-3 study, the median OS of the right side tumor was 23 months and 18.3 months with bevacizumab and cetuximab. For the left side tumor, the median OS with bevacizumab and cetuximab was 28 months and 38.3 months respectively [33]. The difference between the median OS of the left and right side in our cohort was clinically meaningful but did not reach statistical significance (23 and 11 months, CI: 0.48–1.09, *p* = 0.72) probably due to small numbers.

For PFS, on multivariate analysis, high CEA, ECOG > 1 came out to be the predictor for worse outcome. Good performance status has been found associated with better PFS with both single agent and doublet chemotherapy regimens across several studies [22, 34–36]. In NORDIC-VII study, on sub group analysis the PFS benefit with addition of cetuximab was seen in patients with good performance status, KRAS mutant and single metastatic site [37]. On the contrary, the poor prognostic significance of high CEA level at baseline is less conclusive but is documented across in few studies [34, 38].

The study cohort was rich for early-onset colorectal cancer (EOCRC, up to 40 years – 38%). It showed prognostic significance for progression-free survival on univariate analysis only. Existing literature suggests variable prognostic importance [39]. The last four decade has noticed a significant rise in the incidence and mortality of EOCRC globally [3, 40]. EOCRC cancers are comparatively rich in MSI-high status (11–30%), left-sided (73%, especially rectum), risk of synchronous and metachronous tumors, germline mutations(16–35%), signet ring morphology (3–6%), and lynch syndrome (8–18%) [39, 41, 42]. Signet ring morphology and MSI-high status are associated with poor outcomes [39, 42]. In our study cohort, three fourth of signet ring CRC occurred in the EOCRC sub-group. In half of the cases, the primary site was the rectum.

The outcome in our study is inferior than the worldwide literature. The role of biologicals and targeted therapies in improving outcome of metastatic CRC has been well documented. The most likely reason for the inferior outcome compared to developed nations appears to be related to the lack of exposure to subsequent lines of therapy and use of biologicals.

Major limitations of our study is retrospective study design which carries inherent selection bias. Being tertiary care center chances of referral bias was high. The information about the proportion of patients who underwent metastectomy and curative resection was not recorded as very small number (one digit) of patients underwent the procedure. None underwent percutaneous ablation and other local therapies.

## Conclusion

Compared to developed countries, the survival outcome were numerically lower. Well defined prognostic factors (ECOG PS, Albumin, CEA, lines of chemotherapy) were found significant for overall survival. In real word, very few patients got the opportunity to benefit from biologicals, successive lines of chemotherapy and metastasectomy. The study suggest to focus on measures to increase the availability of biologicals through various measures including involvement of patient assistance programme and government assistance schemes. Studies aimed to explore the predictive factors of successful administration of successive lines of therapy are needed. Translational research should be expedited for the distinct epidemiological and clinico-pathological characteristics of colorectal cancer.

## Data Availability

The data sharing was not part of ethical approval and thus currently not available. However if required, data can be shared after approval by ethical committee. A email request can be sent to the corresponding author.

## Abbreviations

CRC: Colorectal Cancer
CEA: Carcinoembryonic Antigen
ECOG: Eastern Cooperative Oncology Group
PFS: Progression Free Survival
OS: Overall Survival
PS: Performance Status
CECT: Contrast Enhanced Computed Tomography
PET CT: Positron Emission Tomography Computed Tomography
ORR: Overall Response Rate
CBR: Clinical Benefit Rate
